# The antihypertensive efficacy of a quadruple single-pill combination in patients with resistant hypertension: study protocol for a randomized, open-label, crossover trial

**DOI:** 10.1186/s13063-025-08719-8

**Published:** 2025-01-20

**Authors:** Yuanyuan Yao, Xin Zhang, Runyu Ye, Shanshan Jia, Xiangyu Yang, Xiaoping Chen

**Affiliations:** Cardiology Department, West China Hospital, Sichuan University, Chengdu, Sichuan Province 610041 People’s Republic of China

**Keywords:** Compound reserpine and triamterene tablets, Resistant hypertension, Spironolactone, Randomized crossover trial

## Abstract

**Background:**

Resistant hypertension (RH) is defined as uncontrolled blood pressure (BP) despite treatment with at least three or more antihypertensive agents. Compelling evidence has shown that such a population has a greater risk of cardiovascular events as well as mortality. Although mineralocorticoid receptor antagonists (MRAs) have been shown to be an effective fourth-line treatment for RH, a significant proportion of RH patients do not achieve their blood pressure target. Compound reserpine and triamterene tablets, a traditional Chinese quadruple single-pill combination, have been proven to have good antihypertensive effects as well as safety, and are promising effective antihypertensive drugs for treating RH.

**Methods:**

A randomized crossover clinical trial will be conducted to compare the efficacy and safety of compound reserpine and triamterene tablets treatment regimen (two tablets of olmesartan/amlodipine (OA) + one tablet of compound reserpine and triamterene tablets) with those of a standard treatment regimen (two tablets of OA + indapamide 2.5 mg + spironolactone 20 mg) in patients with RH. Forty patients will be recruited and randomly assigned in a 1:1 ratio to 2 crossover groups. The two groups will receive different combination therapies for 6 weeks and will then switch to the other combination therapy for 6 weeks, with a 4-week wash-out. The primary outcome will be the reduction in average 24-h systolic blood pressure after 6 weeks of intervention between the two groups.

**Discussion:**

This study aimed to evaluate whether the compound reserpine and triamterene tablets treatment regimen (A + C + 0) results in a greater reduction in blood pressure in RH patients than the standard treatment regimen (A + C + D + spironolactone).

**Trial registration:**

Chinese Clinical Trial Registry ChiCTR2400081878. Registered on March 14, 2024 (http://www.chictr.org.cn).

**Supplementary Information:**

The online version contains supplementary material available at 10.1186/s13063-025-08719-8.

## Administrative information

Note: the numbers in curly brackets in this protocol refer to SPIRIT checklist item numbers. The order of the items has been modified to group similar items (see http://www.equator-network.org/reporting-guidelines/spirit-2013-statement-defining-standard-protocol-items-for-clinical-trials/).

**Table:** 

Title {1}	The antihypertensive efficacy of a quadruple single-pill combination in patients with resistant hypertension: Study protocol for a randomized, open-label, crossover trial
Trial registration {2a and 2b}.	Chinese Clinical Trials Registry; Code: ChiCTR2400081878; Registered on March 14, 2024.
Protocol version {3}	This is the second version of the protocol, finalized on December 6, 2023.
Funding {4}	This trial is supported by the Department of Cardiology, West China Hospital without involvement in study design, collection, analysis and manuscript writing.
Author details {5a}	Department of Cardiology, West China Hospital, Sichuan University, Chengdu, China. Yuanyuan Yao Department of Cardiology, West China Hospital, Sichuan University, Chengdu, China. Xin Zhang Department of Cardiology, West China Hospital, Sichuan University, Chengdu, China. Runyu Ye Department of Cardiology, West China Hospital, Sichuan University, Chengdu, China. Shanshan Jia Department of Cardiology, West China Hospital, Sichuan University, Chengdu, China. Xiangyu Yang Department of Cardiology, West China Hospital, Sichuan University, Chengdu, China. Xiaoping Chen
Name and contact information for the trial sponsor {5b}	West China Hospital Postal Code: 610041 Tell: 028–85422114.
Role of sponsor {5c}	Financial support and supervision.

## Introduction

### Background and rationale {6a}

Resistant hypertension (RH) is treated as a specific phenotype of hypertension defined as BP that remains above target despite the concurrent use of three different antihypertensive agents including long-acting calcium-channel blockers (C:CCB), renin-angiotensin system blockers (A:ACEI/ARB), and diuretics (D) [1]. A recent meta-analysis reported that almost 10.3% of hypertensive patients taking antihypertensive drugs globally suffer from RH [2]. Moreover, RH is likely to be more prevalent in patients with obesity, diabetes, or chronic kidney disease (CKD) [3, 4]. In addition, it is known to be associated with a greater risk of end-organ damage and cardiovascular events [5, 6]. Compared with those with non-resistant hypertension, patients with RH have been demonstrated to have a 32% increased risk of end-stage renal disease (ESRD), a 24% increased risk of ischemic heart event (IHE), a 46% increased risk of congestive heart failure (CHF), and a 6% increased risk of all-cause mortality [5]. The high prevalence and poor prognosis of RH impose a considerable social health burden on society. Therefore, it is essential for us to pay more attention to controlling blood pressure and improving compliance in this population [7].

RH has been shown to be associated with increased blood volume and the activation of the sympathetic nervous system [8, 9]. Excessive intravascular sodium and fluid retention secondary to enhanced aldosterone secretion have been demonstrated to be primary mechanisms [10, 11]. Even if the diagnostic criteria for primary hyperaldosteronism are not met, an elevated low-dose aldosterone status and a low renin phenotype may be important underlying causes of drug resistance in RH [12]. Additionally, marked potentiation of adrenergic activation and baroreflex dysfunction has been reported in the population [13]. Mineralocorticoid receptor antagonists (MRAs), which provide significant benefits in lowering blood pressure in RH patients by antagonizing aldosterone receptors to reduce sodium and water retention, have been recommended as fourth-line treatments for RH [1]. The primary evidence supporting the use of MRAs as a treatment for RH stemmed from the PATHWAY-2 trial. The trial included 335 patients whose blood pressure could not be controlled with a maximal tolerated dose of “A + C + D,” and patients were randomly assigned to receive sequential treatment with spironolactone, bisoprolol, doxazosin, or placebo. The average reduction in home systolic blood pressure caused by spironolactone was 8.7 mmHg greater than that caused by placebo (95% CI was 7.7 ~ 9.7 mmHg), which was the most effective antihypertensive drug during the whole treatment process [14].

However, spironolactone causes hyperkalemia and other adverse effects such as gynecomastia and erectile dysfunction in men and menstrual irregularities in women, which are often difficult for patients to tolerate [1, 15]. Eplerenone, as an alternative to spironolactone, has not been widely used due to its weak efficacy and short half-life [16, 17]. In fact, in a US epidemiological study of RH, only 9.0% of “patients with RH” received MRAs because of adverse reactions, clinical inertia, etc. [18]. Moreover, there was a significant proportion of patients with RH whose BP was still not controlled after treatment with spironolactone [19]. Consequently, it is necessary for clinicians to explore alternatives to spironolactone and simplify the medication regimen for RH.

Compound reserpine and triamterene tablet (Beijing Antihypertensive Number. 0, 0 for short) is a small-dose, multi-ingredient antihypertensive single-pill combination (SPC) innovated and independently developed in China. It consists of 12.5 mg dihydralazine, 0.1 mg reserpine, 12.5 mg hydrochlorothiazide (thiazide diuretic), and 12.5 mg triamterene (potassium-sparing diuretic) and has been confirmed to have a clear antihypertensive effect and good safety [20, 21]. Four antihypertensive drugs, through different mechanisms including sodium excretion, inhibition of sympathetic activity and vasodilation, synergistically exert antihypertensive efficacy and counteract adverse drug reactions. Actually, small doses of potassium-sparing diuretics in combination with thiazide diuretics have been shown to have more pronounced antihypertensive effects than full-dose diuretics alone, while preventing impaired glucose tolerance and hyperkalemia [22]. Moreover, the addition of sympathetic inhibitors such as clonidine or reserpine appears to be an alternative option for patients with RH who cannot tolerate MRAs or who fail to respond to volume-targeting therapies [23, 24]. At the same time, it is well known that patient adherence is inversely proportional to the complexity of treatment and the number of tablets prescribed per day in the management of hypertension [25]. SPC is recommended to be prioritized for treatment in RH to reduce pill burden and improve adherence [26]. As a single-pill combination, compound reserpine and triamterene tablets can increase patient compliance and simplify treatment regimens, while reducing fluid and sodium retention and reducing sympathetic activity, suggesting that it may be an alternative treatment regimen for RH. This study used a prospective, randomized, controlled, open-label, crossover design to compare the difference in the 24-h mean systolic blood pressure reduction between the compound reserpine and triamterene tablets treatment regimen and the standard treatment regimen in RH.

### Objectives {7}

This study investigated the differences in antihypertensive efficacy and safety between the compound reserpine and triamterene tablets treatment regimen (A + C + 0) and the standard treatment regimen (A + C + D + spironolactone) in Chinese patients with RH. This trial will provide a scientific basis for determining whether compound reserpine and triamterene tablets could be used as antihypertensive drugs for treating RH.

### Trial design {8}

This was a randomized 2 × 2 crossover clinical trial that evaluated the antihypertensive effects of compound reserpine and triamterene tablets in RH. To improve patient compliance, we used uniform OA (including 20 mg olmesartan and 5 mg amlodipine), which is a SPC formed by the combination of an angiotensin receptor antagonist and a calcium ion blocker in our study. Considering that small doses of diuretics are already included in the compound reserpine and triamterene tablets, the intervention group contained two tablets of OA + 1 tablet of compound reserpine and triamterene tablets (referred to as A + C + 0). Correspondingly, in the control group, the standard treatment regimen recommended by current international guidelines, two tablets of OA + indapamide 2.5 mg + spironolactone 20 mg (A + C + D + spironolactone for short) were used. We plan to include 40 patients who underwent RH. We have registered the trial at the Chinese Clinical Trials Registry (ChiCTR2400081878). This protocol strictly adhered to the Standard Protocol Items: Recommendations for Interventional Trials (SPIRIT) 2013 statement.

## Methods: participants, interventions, and outcomes

### Study setting {9}

This RCT will be conducted at the West China Hospital of Sichuan University and its three affiliated community hospitals.

### Eligibility criteria {10}

#### Inclusion criteria

1. Age ≥ 18 and < 75 years;
2. Patients with resistant hypertension, that is, after maximal/ maximal tolerated doses of “ACEI /ARB + CCB + D” at the appropriate dosing frequency after one month of continuous treatment, blood pressure does not meet the target, which includes masked uncontrolled hypertension (office BP < 140/90 mmHg and 24 h ambulatory BP ≥ 130/80 mmHg, daytime ambulatory BP ≥ 135/85 mmHg, or nighttime ambulatory BP ≥ 120/70 mmHg) and sustained uncontrolled hypertension (office BP ≥ 140/90 mmHg, 24 h ambulatory BP ≥ 130/80 mmHg, daytime ambulatory BP ≥ 135/85 mmHg, or nighttime ambulatory BP ≥ 120/70 mmHg);
3. Patients can understand the relevant requirements of the study and cooperate with the study intervention;
4. Patients who participate voluntarily and provide written informed consent;

#### Exclusion criteria

1. Secondary hypertension such as primary aldosteronism, Cushing’s syndrome, pheochromocytoma or paraganglioma, renal hypertension, hyperthyroidism, etc.;
2. Malignant hypertension, hypertensive emergency, hypertensive crisis, or hypertensive encephalopathy;
3. Women who plan to become pregnant, pregnant, or lactating;
4. Patients who have a history of allergy to the study drug or its components;
5. Patients with severe organ dysfunction, including impaired renal function (creatinine > 265 µmol/L, estimated glomerular filtration rate（eGFR） < 30 mL/min/1.73 m^2^), bilateral renal artery stenosis, renal artery stenosis with a solitary kidney, hyperkalemia (serum potassium > 5 mmol/L); impaired liver function (aspartate aminotransferase or alanine aminotransferase ≥ 3 times the upper limit of normal); and cardiovascular diseases such as unstable angina, acute coronary syndrome, severe heart failure, life-threatening arrhythmia, and atrial fibrillation;
6. Patients with a history of depression or other psychiatric illness;
7. Patients with active peptic ulcers and ulcerative colitis;
8. Patients who have a history of alcoholism or drug addiction;
9. Medications in use or about to be used will interfere with the results of this study, such as hormones, nonsteroidal anti-inflammatory drugs, immunosuppressants, and other drugs that affect blood pressure;
10. Inability to tolerate ambulatory blood pressure measurement and other circumstances that prevent them from participating in clinical studies.

### Who will take informed consent? {26a}

The research team members will inform eligible individuals about the study details in comprehensible language. Patients who agree to be enrolled will sign an informed consent form.

### Additional consent provisions for collection and use of participant data and biological specimens {26b}

All patient-related data will be used after obtaining individual consent and signing an informed consent form. All personal information in the trial will be anonymized and protected from disclosure.

## Interventions

### Explanation for the choice of comparators {6b}

Spironolactone has been recommended as fourth-line treatment for patients with RH. In order to improve patient compliance and reduce experimental errors, the control group in this trial was standardized to use two tablets of OA + indapamide 2.5 mg + spironolactone 20 mg (A + C + D + spironolactone for short) once a day.

### Intervention description {11a}

**Fig. 1:**
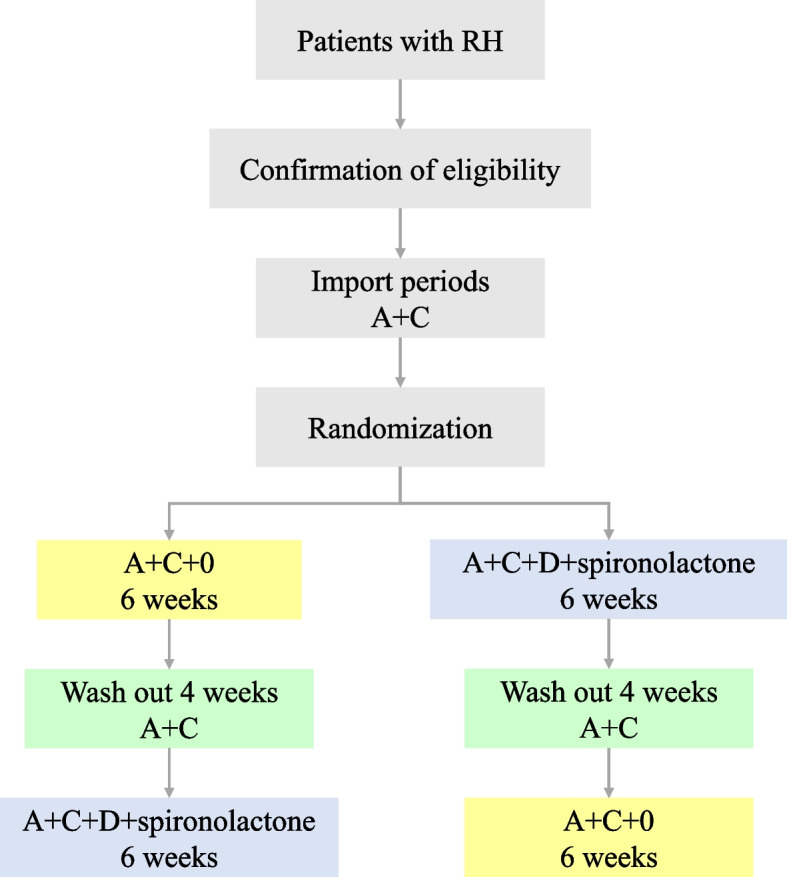
The diagram of this trial

Patients with RH who met the above criteria were included in the study. After a screening visit, patients eligible for randomization proceeded to a 2-week import period with A + C (two tablets of OA daily). After the import period, the participants enrolled will be randomized into two crossover groups at a 1:1 ratio. One group will be given two tablets of OA + 1 tablet of compound reserpine and triamterene tablets (A + C + 0) once a day for 6 weeks, washed with A + C for 4 weeks, and then switched to the standard treatment regimen (A + C + D + spironolactone) for 6 weeks. The other group will be given the standard treatment regimen (A + C + D + spironolactone) for 6 weeks, washed with A + C for 4 weeks, and then switched to A + C + 0 for 6 weeks. We will closely monitor the changes in BP and associated adverse events in the two treatment groups during the two phases. Figure 1 illustrates the diagram of the study.

Determination of the wash-out period: according to the half-lives of the 4 drugs (30–50 h for OA, 14–24 h for indapamide, 9–16 h for spironolactone, and 45–128 h for compound reserpine and triamterene tablets), the wash-out period of this study was 4 weeks, on 3 to 5 half-lives.

In our study, A + C: two tablets of OA (olmesartan 40 mg, amlodipine 10 mg), 0: one tablet of compound reserpine and triamterene tablets, D: indapamide 2.5 mg, spironolactone 20 mg.

### Criteria for discontinuing or modifying allocated interventions {11b}

All patients could voluntarily participate in and withdraw from the study at any time without any reason or consequences. The trial will be discontinued if the patients have a serious adverse reaction.

### Strategies to improve adherence to interventions {11c}

To improve adherence, patients enrolled will be contacted weekly by phone or text messages to remind them to take medication. Research team members will conduct pill counts and help patients complete the eight-item Morisky Medication Adherence Scale (MMSE-8) to assess medication compliance at each face-to-face follow-up visit. Medication compliance = (Number of doses prescribed − Number of doses recovered)/Number of doses prescribed × 100%.

### Relevant concomitant care permitted or prohibited during the trial {11d}

The use of any other antihypertensive medication or medication that affects blood pressure was prohibited during the trial.

### Provisions for post-trial care {30}

All participants will continue to be followed up in hypertension outpatient clinics. If participants suffer any harm from the trial, they will be treated according to the standard medical procedures.

### Outcomes {12}

#### Primary outcomes

The primary objective will be to determine the reduction in average 24-h systolic blood pressure after 6 weeks of intervention between the compound reserpine and triamterene tablets treatment regimen and the standard treatment regimen in RH.

#### *Secondary outcome*s

1. Nighttime and daytime mean ambulatory blood pressure
2. Office blood pressure
3. Morning BP surge in ambulatory blood pressure monitoring (ABPM)
4. BP control rate
5. Anxiety/depression scale scores

#### Safety evaluation

The safety evaluation will include the incidence of adverse events (AEs, defined as any intervention-related unfavorable medical outcomes, including gastrointestinal reactions, hypotension, dizziness, headache, falls, syncope, insomnia, and acute gout attack), serious adverse events (SAEs, including hospitalization, prolonged hospitalization, disability, life-threatening, or death during the trial), and changes in biochemistry results (including electrolyte disturbance, hyperuricemia, and deterioration of kidney function).

The detailed definitions of safety outcomes are as follows:

1. Gastrointestinal reactions, such as bloating, abdominal pain, nausea, vomiting, and belching;
2. Hypotension, defined as SBP < 90 mmHg or DBP < 60 mmHg;
3. Dizziness, defined as a false sense that you or your surroundings are spinning or moving;
4. Headache, defined as a pain in the head or face;
5. Falls, defined as sudden involuntary changes in body position;
6. Syncope, defined as a transient loss of consciousness due to transient global cerebral hypoperfusion characterized by rapid onset, short duration, and spontaneous complete recovery;
7. Insomnia, defined as the condition of being unable to sleep over a period of time;
8. Acute gout attack, defined as a form of arthritis that causes sudden attacks of tenderness and pain in the joints;
9. Electrolyte disturbance, including hyperkalemia (serum potassium > 5 mmol/L), hypokalemia (serum potassium < 3.5 mmol/L), hypercalcemia (serum calcium > 2.75 mmol/L), and hyponatremia (serum sodium < 125 mmol/L), etc.;
10. Hyperuricemia, defined as serum uric acid > 420 μmol/L in men or postmenopausal women, or > 360 μmol/L in women;
11. Deterioration of kidney function, defined as ≥ 50% reduction in eGFR in patients with CKD at baseline, or ≥ 30% reduction in eGFR to < 60 ml/ min/1.73 m^2^ in patients without CKD at baseline, or eGFR < 30 ml/min/1.73 m^2^.

#### Measurement of outcomes

##### 24‑h BP measurement

In this study, ABPM readings were obtained from the nondominant arm using a TM2430 BP monitor (A&D Inc., Tokyo, Japan). Trained nurses will choose suitable size cuffs fitting the participant’s arm circumference to record 24-h BP. Before wearing the cuff, the researchers will explain the precautions to the participants, emphasizing the necessity to maintain their daily routines, avoid vigorous exercise, and remain still during each blood pressure measurement. During monitoring, BP will be measured every 20-min intervals during the day (from 6.00 a.m. to 10.00 p.m.) and every 30 min at night (from 10.00 p.m. to 6.00 a.m.). Reliable ambulatory blood pressure monitoring requires that the number of effective BP readings should reach more than 70% of the total number of monitoring times. Additionally, BP readings should be taken at least once an hour, with at least 20 readings during daytime and at least 7 readings during nighttime. Upon completion of each 24-h recording session, the data were uploaded from each site to the web-based Shuoyun system (https://www.heilcloud.com). Analysis and reporting were conducted in a standardized manner according to the current guidelines by ABPM technologists from West China Hospital.

##### Office BP measurement

Office BP will be measured via calibrated electronic sphygmomanometers (HBP-9020, Omron Corp., Kyoto, Japan). To obtain accurate BP data, all participants were requested to avoid smoking, drinking coffee, or exercising within 30 min. And they will empty their bladder and rest for 5 min in a chair with a backrest before the measurement. BP should be measured in both upper arms at the initial visit, and the side with the higher SBP reading will be the measured upper arm. The measurement of BP should be repeated 1 ~ 2 min apart, and the average of the two readings will be the final BP value. If the two readings of SBP or DBP differ by more than 5 mmHg, a third measurement should be taken and the average of the three readings will be used in the final analysis.

### Participant timeline {13}

See Table 1.

**Table 1:** Schedule of enrolment, interventions, and assessments

Timepoint	-2 week	0 week	6th week	10th week	16th week
Eligibility screen	×				
Informed consent	×				
Demographic data	×				
Medical history	×				
Physical examination	×	×	×	×	×
Biochemistry		×	×		×
24 h ambulatory blood pressure monitor	×	×	×	×	×
GAD-7 scores		×	×	×	×
PHQ-9 scores		×	×	×	×
Concomitant medications	×	×	×	×	×
Compliance with medication		×	×	×	×
Adverse event record		×	×	×	×

• Demographic data: name, sex, ID number, date of birth, telephone number, race, occupation, marital status and history of drug allergies

• Medical history: history of cardiovascular and cerebrovascular disease, history of obstructive sleep apnea syndrome, history of smoking or passive smoking, alcohol abuse, and medication use

• Physical examination: office BP, height, weight, waist circumference, heart rate, and respiratory rate

•Biochemistry: blood routine, renal function, liver function, blood lipids, electrolytes, fasting blood glucose, and urine routine

• GAD-7: Generalized Anxiety Disorder 7-Item Scale

• PHQ-9: Patient Health Questionnaire-9

### Sample size {14}

To assess the noninferiority of the intervention group to the standard treatment regimen in terms of the primary efficacy outcome, the margin of noninferiority was set to 2.5 mmHg in the current study. Considering the differences between individuals and the allowable error of blood pressure monitoring, a blood pressure difference of 5 mmHg is common in clinical settings. To be conservative, after discussion with clinical experts, half of the 5 mmHg was adopted as the margin in the current study. Few studies have evaluated the antihypertensive efficacy of spironolactone versus compound reserpine and triamterene tablets in the same trial in patients with RH. Hence, we empirically estimated a difference of 5 mm Hg in systolic BP between treatment groups with a standard deviation SD of 15 mm Hg. The power is set at 80% (one-sided alpha level of 0.025) in the trial. We use the following formula, which is specially for sample size calculation of crossover noninferiority design to calculate the total number. The result is *n* = 16. We also calculate the sample size via PASS version 15.0, with the sample size of *n* = 17. Considering 15% loss to follow-up, a final sample size of 40 participants with 20 in each crossover group is requested in the trial.

$$n=\frac{{\left({Z}_{1-\alpha }+{Z}_{1-\beta }\right)}^{2}{{\sigma }_{m}}^{2}}{2{\left(\upvarepsilon -\Delta \right)}^{2}}$$

- *n* = sample size in each group
- *α* = type 1 error = 0.025
- *β* = 1-power = 0.2
- ${\sigma }_{m}$ = the standard deviation of the difference in SBP reduction between the two treatment groups
- $\varepsilon$ = the difference between mean systolic blood pressure values of the two treatment groups
- $$\Delta$$ = noninferiority cutoff ($$\Delta$$<0)

### Recruitment {15}

Potential patients with RH will be identified from the hypertension outpatient clinics and inpatient department. We will invite them to participate by phone or face-to-face communication.

## Assignment of interventions: allocation

### Sequence generation {16a}

An independent statistician will use a computer-generated sequence of random numbers to determine whether participants are enrolled in the intervention or control group.

### Concealment mechanism {16b}

Sequential numbers will be placed in sealed envelopes to assign eligible patients to either the intervention or control group. The order of grouping will be kept confidential.

### Implementation {16c}

The allocation sequence will be created by an independent statistician from the main research unit, whose task is only to perform randomization according to the study protocol. He/she will not be involved in patient recruitment, follow-up, data collection, or analysis.

## Assignment of interventions: blinding

### Who will be blinded {17a}

Due to the different dosage forms of the drugs in the two groups, it is not feasible for patients to be blinded. Additionally, the attending physicians cannot be blinded because they need to dispense medications as well as complete patient follow-ups. However, except for patients and the attending physicians, all others (including nurses, clinical investigators, coordinators, data managers, data monitoring committee (DMC), ABPM technologists, and statisticians) will be blinded to patient grouping and drug assignment.

### Procedure for unblinding if needed {17b}

Considering that patients and the attending physicians are not involved in blinding, so unblinding will not occur.

## Data collection and management

### Plans for assessment and collection of outcomes {18a}

In the trial, all research team members will read and understand the content of the trial protocol in detail and strictly follow the protocol arrangement to complete clinical visits. Trained nurses, clinical research coordinators (CRCs), and data managers are required to maintain medical records and collect data in our study. Before the import period, participants’ demographic data, medical history, and BP data will be collected. At the end of 2-week import period and during the 6th, 10th and 16th week of follow-up, we will collect patients’ office BP, 24-h ABPM data, and assess patients’ anxiety and depression levels. Biochemistry data will be collected during 6th and 16th week of follow-up.

### Plans to promote participant retention and complete follow-up {18b}

The participants will be contacted weekly by phone or text messages. And 3 days prior to the face-to-face consultation, we will make a telephone appointment with the participants to schedule the date and time of the follow-up visit.

### Data management {19}

The research team members will accurately, timely, and legally collect the medical records (photos and videos) and complete the electronic case report form (CRF) on the electronic data capture system, namely, the Red Shine Chronic Disease Management System developed by the Hypertension Center at West China Hospital.

### Confidentiality {27}

All the data will be entered into the Red Shine Chronic Disease Management System. All patient-related information in this trial will be restricted to the principal investigator and specific research team members.

### Plans for collection, laboratory evaluation, and storage of biological specimens for genetic or molecular analysis in this trial/future use {33}

Not applicable. Biological samples obtained from individuals will be used only to evaluate the results of this trial and will not be used for genetic or molecular analysis.

## Statistical methods

### Statistical methods for primary and secondary outcomes {20a}

For descriptive statistics of the baseline data, the mean ± standard deviation was used for normally distributed continuous variables, the median and interquartile ranges were used for nonnormally distributed continuous variables, and the frequency and percentage were used for dichotomous variables. For comparisons between two groups, quantitative variables were compared via the *t*-test or rank-sum test, and qualitative variables were compared using the chi-square test or Fisher’s exact test. The primary objective of the study (change in 24-h mean SBP) will be analyzed using a linear mixed-effects model that will include all prespecified covariates (including baseline BP, age, sex, body mass index, alcohol consumption status, smoking status, fasting glucose, total cholesterol, low-density lipoprotein cholesterol, etc.) to analyze the treatment effect, stage effect, and sequence effect. The linear model will also be used to evaluate the other continuous variables of secondary outcomes. Categorical variables of secondary outcomes and safety outcomes will be analyzed by the chi-square test and Fisher’s exact probability method. We will use the generalized estimating equation (GEE) model to compare the changes in qualitative variables between the two groups. All the statistical analyses will be performed via SPSS version 26.0 and R software version 4.2.1. The significance level will be set to 5% for all the statistical tests.

### Interim analyses {21b}

The trial did not include an interim analysis. If frequent adverse effects are detected, the intervention will be discontinued and a formal report will be submitted to the Ethics Committee of West China Hospital for their decision-making.

### Methods for additional analyses (e.g., subgroup analyses) {20b}

In this trial, no additional analyses are intended to be conducted.

### Methods in analysis to handle protocol non-adherence and any statistical methods to handle missing data {20c}

All outcomes will be statistically analyzed according to the intention-to-treat (ITT) principle and the per-protocol (PP) population separately. The process of handling missing data is generally divided into deletion or imputation of missing data. The statistician will use different methods, such as the mean imputation, last observation carried forward, and multiple imputation, to address missing data depending on the specific circumstances.

### Plans to give access to the full protocol, participant level-data, and statistical code {31c}

Upon reasonable request, the corresponding author can provide complete protocol information as well as relevant data.

## Oversight and monitoring

### Composition of the coordinating centre and trial steering committee {5d}

The Ethics Committee and the principal investigator will oversee and coordinate all phases of the study. They do not have any conflicts of interest.

### Composition of the data monitoring committee, its role, and reporting structure {21a}

This trial will establish an independent data monitoring committee (DMC) consisting of three members experienced in cardiology. The role of the DMC is to conduct an unbiased review of suspected clinical endpoints and adverse events, protect the safety of the participants, and ensure the validity of the data.

### Adverse event reporting and harms {22}

If the subject has serious adverse events or other unpredictable risks, the attending physicians will report them in a timely manner, and the DMC will immediately take appropriate treatment measures and decide whether to deviate from the clinical trial plan. In any emergency condition, it is necessary to protect the interests of the subjects.

### Frequency and plans for auditing trial conduct {23}

The research team will hold a meeting once a week to discuss patient enrollment and follow-up, adverse event data, and needed protocol adjustments.

### Plans for communicating important protocol amendments to relevant parties (e.g., trial participants, ethical committees) {25}

Any modifications to the protocol will be submitted to the Ethics Committee of West China Hospital and the Chinese Clinical Trial Registry for approval. The enrolled participants will be informed of these changes and their possible effects. Protocol modifications will be updated at ClinicalTrials.gov.

### Dissemination plans {31a}

This research protocol and results will be published as an article in an international journal for peer review. Other interested researchers can refer to the data from this experiment for further studies.

## Discussion

The purpose of this study is to evaluate the efficacy and safety of compound reserpine and triamterene tablets in resistant hypertension. RH has been associated with inappropriately elevated aldosterone levels and activation of the sympathetic nervous system. Although MRAs are recommended by most guidelines as a fourth-line treatment option, the BP control rates remain low in patients with RH. Moreover, there are no studies showing a clear benefit of MRAs on outcome events in patients with RH. Compound reserpine and triamterene tablets, having synergistic effects of reducing sodium retention and sympathetic nerve impulses, contribute to a relatively “simplified” regimen of RH to improve adherence and could be promising alternative drugs for treating resistant hypertension.

### Limitation

This study has several limitations. Firstly, our study included only Chinese RH individuals, so the findings may not be applicable to other ethnic groups. Secondly, the study did not standardize the number of tablets, appearance, or color between the two treatment regimens, which may affect patients’ medication adherence. Thirdly, this trial only followed patients for short-term blood pressure reductions; therefore, cardiovascular outcomes could not be assessed in the longer term.

### Trial status

The trial was registered on ClinicalTrials.gov on March 14, 2024, which is the second version of the protocol. This trial is ongoing, which the recruitment began in June 2024 and is expected to end approximately November 2024.

## Supplementary Information

Supplementary Material 1.

## Data Availability

The datasets used and/or analyzed after the completion of this study are available from the corresponding author upon reasonable request.

## Abbreviations

RH: Resistant hypertension
BP: Blood pressure
OA: Olmesartan/amlodipine
ACEI: Angiotensin-converting enzyme inhibitors
ARB: Angiotensin II receptor blockers
CCB: Calcium-channel blockers
D: Diuretics
ESRD: End-stage renal disease
IHE: Ischemic heart event
CHF: Congestive heart failure
MRAs: Mineralocorticoid receptor antagonists
SPC: Single-pill combination
MMSE-8: 8-Item Morisky Medication Adherence Scale
ABPM: Ambulatory blood pressure monitoring
ADRs: Adverse drug reactions
SAEs: Serious adverse events
GAD-7: Generalized Anxiety Disorder 7-Item Scale
PHQ-9: Patient Health Questionnaire-9
CRC: Clinical research coordinator
CRF: Case report form
DMC: Data monitoring committee
